# Cognitive penetrability of scene representations based on horizontal image disparities

**DOI:** 10.1038/s41598-022-22670-7

**Published:** 2022-10-25

**Authors:** Yulan D. Chen, Milena Kaestner, Anthony M. Norcia

**Affiliations:** 1 Department of Psychology, Stanford University, 450 Jane Stanford Way, Stanford, CA USA; 2 Wu-Tsai Neuroscience Institute, Stanford University, 290 Jane Stanford Way, Stanford, CA USA

**Keywords:** Attention, Visual system, Object vision

## Abstract

The structure of natural scenes is signaled by many visual cues. Principal amongst them are the binocular disparities created by the laterally separated viewpoints of the two eyes. Disparity cues are believed to be processed hierarchically, first in terms of local measurements of absolute disparity and second in terms of more global measurements of relative disparity that allow extraction of the depth structure of a scene. Psychophysical and oculomotor studies have suggested that relative disparities are particularly relevant to perception, whilst absolute disparities are not. Here, we compare neural responses to stimuli that isolate the absolute disparity cue with stimuli that contain additional relative disparity cues, using the high temporal resolution of EEG to determine the temporal order of absolute and relative disparity processing. By varying the observers’ task, we assess the extent to which each cue is cognitively penetrable. We find that absolute disparity is extracted before relative disparity, and that task effects arise only at or after the extraction of relative disparity. Our results indicate a hierarchy of disparity processing stages leading to the formation of a proto-object representation upon which higher cognitive processes can act.

## Introduction

Surfaces and objects in natural scenes can be distinguished based on gradients and discontinuities in a wide range of local features and the coding of gradients and discontinuities involves non-local feature measurements. The earliest stages of so-called figure-ground segmentation have been extensively studied for motion^1–6^ and orientation discontinuities^7–11^ in V1 and V2 of macaque where sensitivity to discontinuities in these features is present.

Discontinuities in binocular disparity also provide robust signals for figure ground segmentation and can do so in the absence of any other cues^12^. Binocular disparity is an interesting case, because unlike the case of motion and orientation discontinuities, where both local cues themselves and discontinuities in these cues are robustly signaled in V1, only local disparities—so-called absolute disparities—are robustly represented in V1, with the representation of disparity discontinuities (relative disparities) being deferred until V2^13–16^. Importantly, cells in V1 are tuned for the absolute disparity of both correlated and anti-correlated stereograms^17^. Only the former support a percept of depth. Thus, the neural basis of perceptual depth must lie outside of V1^18^.

**Figure 1:**
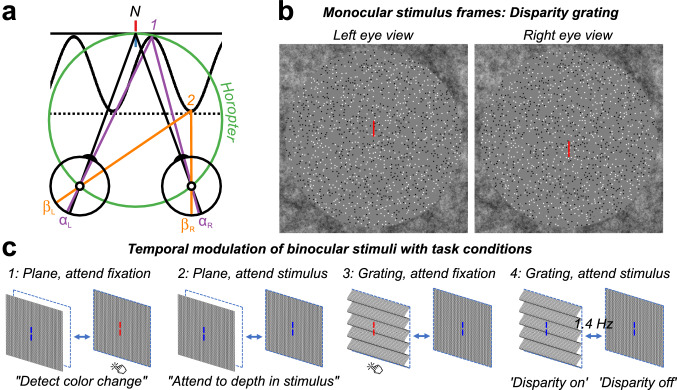
(**a**) Top-down schematic illustration of absolute and relative disparity. Fixation point N (nonius lines) is on the zero-disparity plane defined by the horopter (green Vieth-Muller circle). Point 1 (purple) is also on the horopter and angles ${\mathrm{\alpha }}_{\mathrm{L}}$ and ${\mathrm{\alpha }}_{\mathrm{R}}$ are equal, meaning that the absolute disparity given by ${\mathrm{\alpha }}_{\mathrm{L}}- {\mathrm{\alpha }}_{\mathrm{R}}$ is zero. Point 2 (orange) is either on a second plane (dotted line) or on the peak of a disparity grating (sinusoidal line). At point 2, the absolute disparity, ${\upbeta }_{\mathrm{L}}- {\upbeta }_{\mathrm{R}}$, is non-zero. The relative disparity between points 1 and 2 is the difference between the two absolute disparities, (${\mathrm{\alpha }}_{\mathrm{L}}- {\mathrm{\alpha }}_{\mathrm{R}})-({\upbeta }_{\mathrm{L}}- {\upbeta }_{\mathrm{R}}).$ In contrast to the absolute disparity, the magnitude of the relative disparity is independent of fixation. (**b**) Random-dot stereopair used in the main experiment depicting a sinusoidal disparity grating (crossed disparity when cross-fused). The dots in the actual experiment were dynamic. (**c**) Schematic of different stimuli, task conditions, and temporal modulation between crossed disparity, ‘disparity on’, and zero disparity, ‘disparity off’ phases. Participants were asked to detect a brief color change on the fixation lines, or to attend to the depth modulation of the stimulus, for either plane (absolute disparity only) stimuli or grating (absolute and relative disparity) stimuli. To generate a visual-evoked potential, all stimuli modulated between disparate and non-disparate states at 1.4 Hz.

In a parallel line of research, the human psychophysical and oculomotor literatures have also suggested that absolute disparity representations are perceptually inaccessible. When confronted with a large, uniform random-dot stereogram whose absolute disparity is slowly changed over time, observers are unaware of the motion in depth^19,20^, even under stabilized image conditions that produced large absolute disparities that could not be compensated by vergence eye movements^21^. The addition of a disparity reference, creating relative disparity cues, greatly increased the percept of motion-in-depth. Stimuli containing vertical absolute disparity also evoke no percept of motion-in-depth, but can drive vergence^22^. A comparison of the relationships between absolute and relative disparity thresholds and vergence noise has suggested that absolute disparity, per se is perceptually inaccessible, something the authors termed the absolute disparity anomaly^23^. The geometric definitions of absolute and relative disparities are illustrated in Fig. 1a.

If absolute disparities are perceptually inaccessible, their processing should be relatively immune to the observer’s task. By contrast, responses to perceptually relevant relative disparities may be subject to task effects. The literature on orientation-based figure-ground processing has posited a significant role for top-down inputs to the figure-ground process and further suggests a time-course for attentional modulation relative to the extraction of local features and local feature relationships. V1 cell responses of macaque in an orientation-defined form task show that the effect of task occurs after orientation discontinuities and by implication orientation tuning per se was established^24^. Later work^25^ suggested a model comprised of an initial feature registration stage, here local orientation, that was followed by detection of the feature-difference boundary, which was then followed by attention-dependent enhancement of the figure region and later suppression of the background. In related work^26^, border-ownership relations in a display involving separated or occluding figures showed that some cells signaled border ownership independent of attention, but that in a substantial number attention acted after border ownership was established in the case of separated figures and at the same time as border ownership was established of occluded figures.

Task effects on neural responses to absolute and relative disparity cues have not been studied before, but there are clear predictions for what should be observed from the existing literature where absolute disparity processing should be relatively immune from the effects of task. The extent to which relative disparity coding is influenced by task is a more open question. Here, we compared 128-channel evoked Visual Evoked Potentials (VEPs) to dynamic random-dot stereograms (DRDS) that either portrayed a flat surface moving in depth (changing absolute disparity) or a flat surface changing to a corrugated one (changing absolute and relative disparity). For readers who can free-fuse stereograms, a crossed-disparity (static) random-dot stereogram depicting a disparity grating is shown in Fig. 1b. The use of DRDS stimuli allowed us to isolate disparity-related responses from responses due to monocular stimulation, and the use of the grating vs. plane stimuli allowed us to manipulate the availability of relative disparity information. To modulate potential top-down influences on these responses, we recorded under task conditions in which the disparity modulation was either task relevant (‘attend stimulus task’) or task-irrelevant (‘attend fixation task). Schematic illustrations of stimuli, temporal dynamics, and task conditions are illustrated in Fig. 1c.

We find that the initial evoked response to disparity change is the same for the disparity plane and disparity grating stimuli, suggesting that the leading edge of the response reflects the processing of absolute disparity information that is common to the two stimulus conditions. We also find that the response to the disparity grating condition is more affected by task, and that this task modulation occurs at or after the extraction of relative disparity.

## Results

### Topography of disparity responses

**Figure 2:**
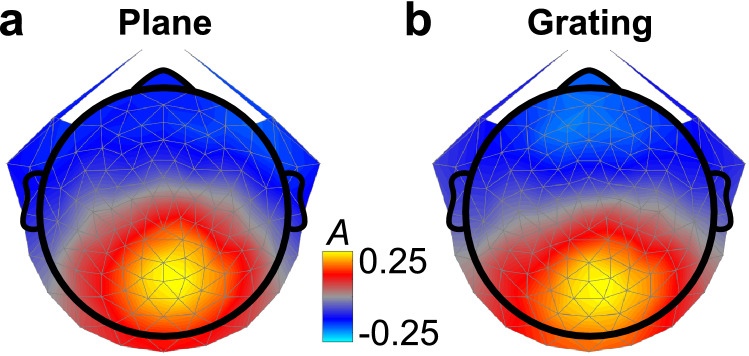
Topographies of the first reliable component for responses to plane and grating stimuli (N = 20). Units of the forward model (A) were maximal over midline occipital electrodes, indicating that neurons in early visual cortex were responding systematically across trials. Small differences between the plane (**a**) and the grating (**b**) responses include that the grating response is shifted more towards posterior electrodes and is broader.

We used a spatial filtering approach (Reliable Components Analysis, RCA; see Methods: VEP Signal Processing and Reliable Components Analysis, also Fig. 7b) to reveal clusters of electrodes that responded in a systematic manner across stimulus presentation trials^27^. RCA improves SNR and reduces the dimensionality of our 128-channel electrode data into a smaller number of components, whilst also revealing underlying, physiologically plausible neural sources that are illustrated in Fig. 2. Here, we show the topographies of the first reliable component, RC1, highlighting a cluster of midline occipital electrodes that respond reliably across trials of the plane (a) or the grating (b) stimulus. Sensor-space weights were learned on ‘nF1 clean’ filtered data in the time domain (see Fig. 7a(iii)), collapsing across task conditions (‘attend fixation’ and ‘attend stimulus’) but not between disparity stimulus types (plane vs. grating). The resulting topographies for the first component RC1, shown in Figure, 2 were produced via a forward model generated from the learned weight vectors^27,28^ and are visually similar, and consistent with neural sources in V1, V2, V3 and V3A^29–32^. We note that the response to the grating is somewhat more diffuse, commensurate with a more widespread neural response to relative disparities. In the remainder, we present results from these topographies, where 128-channel data from each condition were weighted by either the ‘plane’ or the ‘grating’ RC1 vector to generate group-level waveforms and error estimates.

### Time-course of disparity plane and grating responses

**Figure 3:**
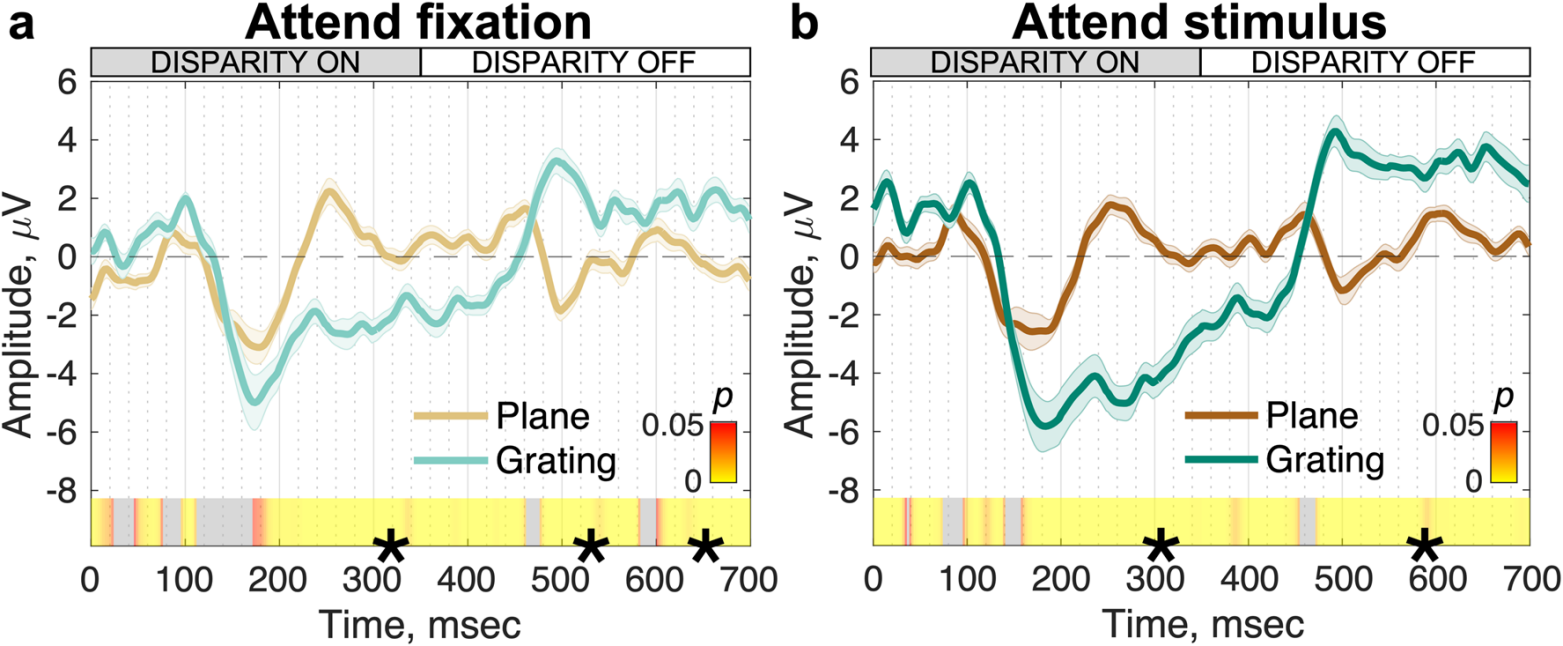
Cycle averages of VEP responses to disparity changes for plane and grating stimuli under ‘attend fixation’ (**a**) and ‘attend stimulus’ (**b**) task conditions (N = 20). In both panels, VEP waveforms are reconstructed via the nF1 clean filter and are weighted by the first reliable component (topographies in Fig. 2). Waveforms are group-level mean responses to a single stimulus cycle consisting of one ‘disparity on’ transition at 0 ms and a ‘disparity off’ transition at ~ 357 ms. Errors are $\pm$ 1 SEM. The heat bar indicates the p-value significance of the plane (brown colors) vs. grating (green colors) responses calculated via permutation-based paired *t*-tests. Segments marked with (*) indicate sequential, significant time points that pass the threshold for run-length correction and grey = nonsignificant.

To assess the relative timing of plane and grating disparity processing, we first compare responses of the disparity plane to the disparity grating during the nonius color-change task (Fig. 3a). The disparity-evoked responses both show a positive peak at ~ 100 ms post-disparity onset and diverge significantly from one another at 172 ms on the downward slope of a negative-going potential, where this deflection is larger for the relative disparity condition. Notably, the response to the disparity grating is sustained for the duration of the disparity-on phase, whilst the response to the disparity plane is transient at the disparity-on and disparity-off transitions. Differences in response profile drive the long period of significant differences between the waveforms post 172 ms. For the phase of significance between 172 and 461 ms, the mean Bayes factor (${bf}_{10}$) is 1.32 $\times$ 10^4^—extreme evidence for ${H}_{1}$^33,34^. Interestingly, the initial portion of the disparity response (~ 100–172 ms) does not differ between plane and grating conditions after run-corrected permutation testing. There is a brief period of significance that does not pass run-length correction at around 100 ms. The Bayes factor (${bf}_{10}$) during this phase (~ 100–172 ms) is much lower at 7.87. According to the evidence categories laid out in^33,34^, this amounts to moderate evidence for ${H}_{1}$. However, in conjunction with the run-length correction, the extremely high Bayes factor values we measure at other significant time intervals, as well as the comments by^34^ on the cautious interpretation of Bayes factors in scientific research, we do not consider this to be sufficient evidence against the null hypothesis (see Methods for further discussion).

We also compared responses to the two disparity types under ‘attend stimulus’ conditions (Fig. 3b). Here the disparity-evoked responses also show positive peaks at ~ 100 ms, but they diverge at 158 ms (${bf}_{10}$ between 158 and 454 ms = 1.17 $\times$ 10^5^ ; extreme evidence for ${H}_{1}$^33,34^) rather than at 172 ms in the attend fixation condition. Using the Jackknifing procedure described in the Methods, we determined the uncertainty around these points of divergence, and found that the time at which the responses to the plane and grating stimuli diverge is significantly later when attending to fixation (median Jackknifed ‘attend fixation’ divergence estimate = 172.96 ms ± 21.02 ms) versus attending to the stimulus (median Jackknifed ‘attend stimulus’ divergence estimate = 157.45 ms ± 5.70 ms; *t(*19) = 15.44, *p* < 0.001, ${bf}_{10}$= 2.25 × 10^9^; extreme evidence for ${H}_{1}$^33,34^).

Asking the observers to attend to the changing disparity stimulus rather than to the nonius lines can thus speed up the differentiation of the plane stimulus that contains only absolute disparity, from the grating stimulus which contains both absolute and relative disparity, but not to the extent that it eliminates the common initial positive/negative deflections.

### Does task have equivalent effects on plane and grating responses?

**Figure 4:**
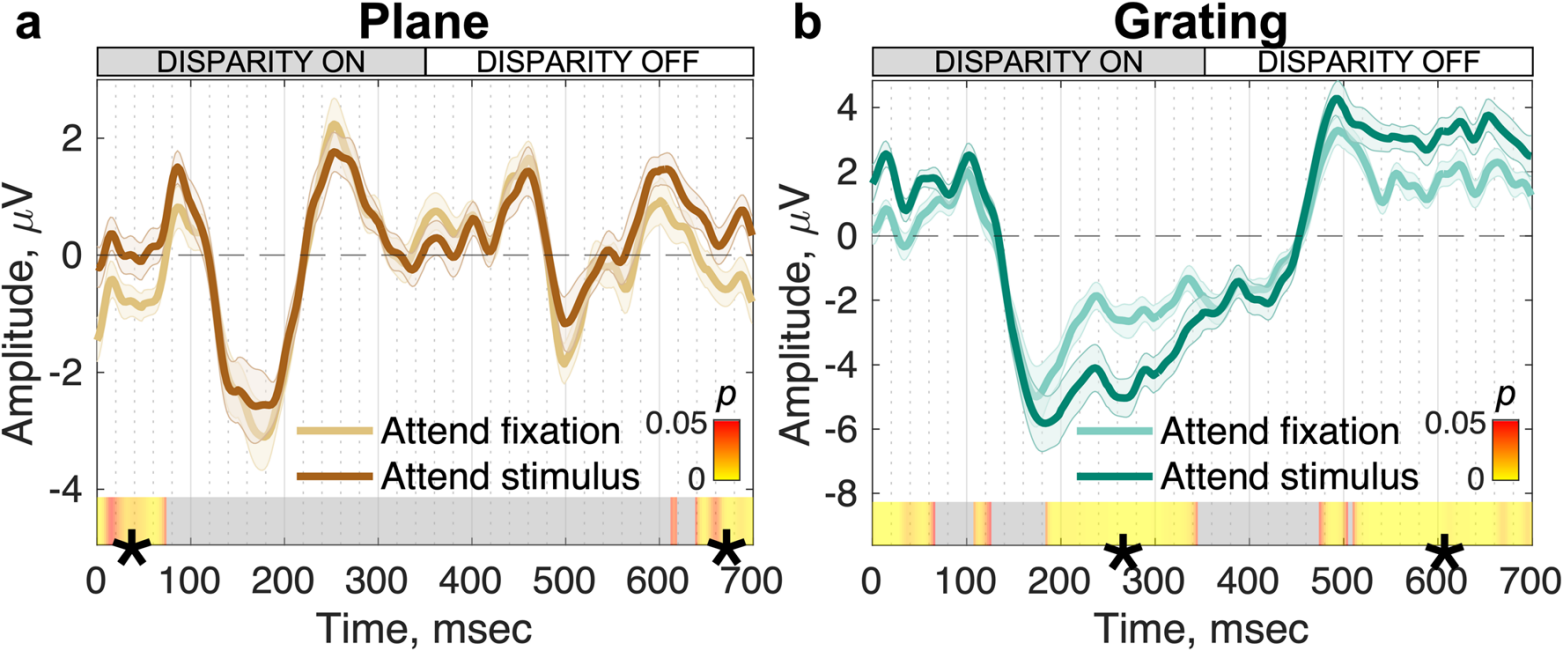
EEG responses to plane and grating disparity stimuli under different task demands (N = 20). (**a**) Responses to the plane (pure absolute disparity) stimulus. Responses during attend fixation (light brown) and attend stimulus (dark brown) conditions are highly similar and transient waveforms are observed at both disparity on and disparity off stimulus transitions. (**b**) Responses to the grating (relative and local absolute disparity) stimulus. Responses during the attend fixation (light green) and attend stimulus (dark green) are initially similar up to ~ 184 ms when they begin to diverge. For both panels, data are the group mean waveform in RC1, and errors are $\pm$ 1 SEM. Data have been filtered using the nF1 clean filter. Heat bars indicate significance (paired-samples *t*-test) and segments marked with (*) indicate sequential, significant time points that pass the threshold for run-length correction and grey = nonsignificant.

We asked whether the plane and grating conditions are equally affected by task. As shown in Fig. 4, responses to the plane condition under the two tasks are similar, overlapping entirely during the two transient phases of the response that occur at disparity-on and disparity-off transitions (negative peaks at 176 ms and 498 ms for both task conditions). The mean Bayes factor (${bf}_{10}$) between 76 and 635 ms (the period of non-significance) was 0.44; anecdotal evidence for ${H}_{0}$^33,34^. The brief periods of significance at the start and end of the cycle are likely driven by residual wrap-around effects of later activity (for further discussion see below) and occur before and after stimulus-evoked activity associated with the disparity change; here, ${bf}_{10}$ was 7.15 and 7.85, respectively, which is considered moderate evidence for ${H}_{1}$^33,34^.

Grating stimuli, as opposed to plane stimuli, contain more perceptually relevant information with the addition of relative disparity cues, and thus we expected to find greater effects of task here. As was the case for the plane disparity conditions, the leading edge of the responses for the grating stimuli are identical (102–184 ms); here, the ${bf}_{10}$ = 1.19, which is considered anecdotal evidence for ${H}_{1}$^33,34^—but see our discussion on the interpretation of Bayes factors in conjunction with permutation *t*-testing in the Methods. However, the ‘attend stimulus’ condition results in a larger negative-going deflection that is sustained throughout the ‘disparity on’ phase of the stimulus (Fig. 4b). The magnitude of the response significantly differentiates the two task conditions, starting at 184 ms after disparity onset and lasting through 344 ms (${bf}_{10}$ = 64.03, very strong evidence for ${H}_{1}$^33,34^). This effect occurred after the differentiation of the plane and grating responses at 158 or 172 ms, depending on attentional demands (Fig. 3), and Jackknifing the onset of the effect revealed that this difference was significant in both cases (median Jackknifed ‘grating’ divergence estimate = 183.69 ms ± 5.57 ms; *t*(19) = -7.17, *p* < 0.001, ${bf}_{10}$= 2.16 × 10^4^10^21^—extreme evidence for ${H}_{1}$^33,34^—for the comparison against the ‘attend fixation’ divergence estimate; *t*(19) = 25.29, *p* < 0.001, ${bf}_{10}$= 6.79 × 10^21^—extreme evidence for ${H}_{1}$^33,34^—for the comparison against the ‘attend stimulus’ divergence estimate). Task effects are also seen at comparable latencies after disparity offset. Although our task manipulation resulted in large signal deflections later in the stimulus cycle, it did not affect the initial phase of the response at disparity-onset.

### Separating transient and sustained components via spectral filtering

**Figure 5:**
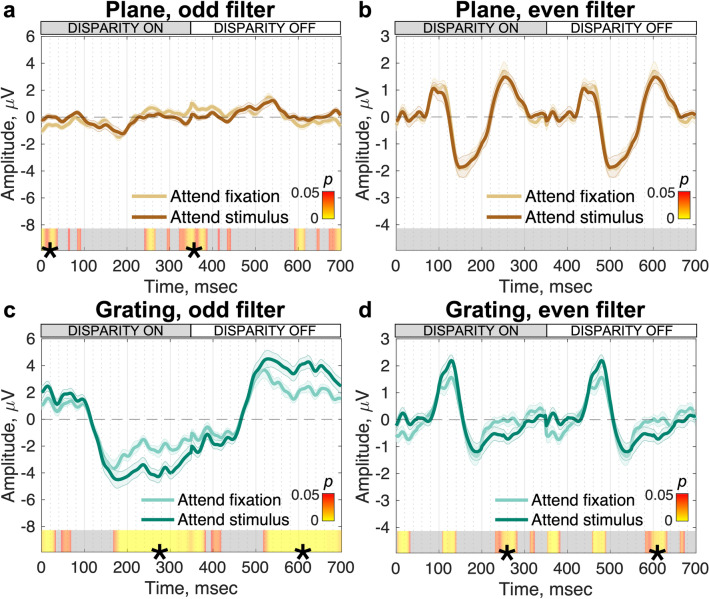
Task effects on EEG responses to disparity stimuli under different Fourier filtering regimes that highlight sustained vs. transient response components (N = 20). (**a**) and (**b**) show responses to the plane stimulus (absolute disparity); **c** and **d** show responses to the grating stimulus (relative and local absolute disparities). The “Odd” filter (left column) preserves the odd harmonics of the fundamental frequency (1F1, 3F1, 5F1, etc.) and highlights sustained response components driven by the amplitude of the 1F1 response. Where this signal is present (**c**), there is a significant effect of task. The “Even” filter (right column) preserves the even harmonics of the fundamental frequency (2F1, 4F1, 6F1, etc.) and reveals transient, temporally symmetric responses at disparity on- and off-sets. There is no task effect for the plane stimulus (**b**) and there are some small effects for the grating (**d**), but these are localized outside the main positive/negative deflections that demarcate the stimulus transitions. Data are group-level averages weighted by RC1, errors are $\pm$ 1 SEM and the color bars indicate significance (paired-samples *t*-test). Segments marked with (*) indicate sequential, significant time points that pass the threshold for run-length correction and grey = nonsignificant.

**‘**Odd’ and ‘even’ filters in the Fourier domain can be used to reveal transient and sustained response components that have been associated with the extraction of plane and grating disparity signals respectively^35^. The logic of the filtering is based on prior work with contrast responses^36^. Even harmonics capture transient activity that is the same after either disparity on/off or off/on transitions, while the odd harmonics, and especially the first harmonic, capture sustained activity that differs after the disparity off/on (plane to grating) vs on/off (grating to plane) transitions. The transient response component to the grating stimulus was separated by reconstructing the waveform using only even harmonic response components (2F1, 4F1, 6F1, etc.), and the sustained component using only odd harmonic components (1F1, 3F1, 5F1, etc.). Filtering further highlights the differences in the response profiles for the grating and the plane conditions, where the response to the plane is dominated by even harmonics (Fig. 5b) whilst the response to the grating is contains both odd (Fig. 5c) and even (Fig. 5d) harmonic responses. For the grating stimuli, odd harmonic responses are at least a factor of two larger in amplitude than even harmonic responses. Thus, the plane stimulus, containing only absolute disparities, drives an even symmetric response with transient on and off responses, whilst the grating stimulus, containing both absolute and relative disparities, drives a response with both odd and even harmonic components. This pattern of results associates the odd harmonics with the processing of relative disparity.

How does the filtering approach reveal effects of task? For the plane stimulus, the even-filtered response shows no measurable effect of task (${bf}_{10}$ = 0.29, anecdotal evidence for ${H}_{0}$^33,34^) and a small odd-harmonic response with task effects at the edges of the cycle average (${bf}_{10}$ = 3.55, moderate evidence for ${H}_{1}$^33,34^; Figs. 5a and 5b). The odd harmonic response in the grating condition is by contrast ~ 8 times larger than in the plane condition (compare Figs. 5a and 5c) and shows substantial effects of tasks, starting at 167 ms (Fig. 5c) —17 ms earlier than in the unfiltered data (median ‘odd’ filter divergence estimate for grating stimulus = 169.38 ms ± 16.44 ms; Jackknifed *t*-test to compare against ‘grating’ divergence estimates, *t*(19) = -19.37, *p* < 0.001, ${bf}_{10}$ = 1.32 × 10^9^—extreme evidence for ${H}_{1}$^33,34^). As in the case of the unfiltered data, there is an initial period of response that is not modulated by task between 100 and 167 ms (${bf}_{10}$ = 0.58, anecdotal evidence for ${H}_{0}$^33,34^). The even-filtered response to the grating condition (Fig. 5d) has measurable effects of task at 230–280 ms (${bf}_{10}$ = 5.07, moderate evidence for ${H}_{1}$^33,34^) after a period between 75 and 200 ms where the disparity-specific response is present but not affected by task (${bf}_{10}$ = 3.46, anecdotal to moderate evidence for ${H}_{1}$^33,34^ when including a brief period of significance that does not pass run-length correction, see our discussion in the Methods on interpretation of the Bayes factor in these instances). The onset of the earliest disparity specific response is now more clearly defined in the even filtered responses, and occurs at ~ 75 ms for both the disparity plane and grating conditions.

## Discussion

By using a combination of high-density EEG, a spatio-temporal decomposition approach, and behavioral task manipulations we have mapped out the temporal order of the early stages of disparity processing and their modulation by task. We found that during the earliest time-points after disparity onset, responses to the plane and grating conditions, and by implication responses to absolute and relative disparity information, did not differ from each other and were not dependent on task. We also observed a brief period where relative disparity information was registered independent of task after which the effect of task manifested primarily on responses we associate with the extraction of relative disparity. In the following we detail the timeline of disparity processing with respect to our own data and to the broader context of models of attentional influences on scene processing.

### Time-courses for the onset of absolute and relative disparity processing

DRDS stimulation isolates disparity-specific responses as the stimuli contain no monocular cues for the disparity changes that elicit the evoked response. By varying the spatial structure of our stimuli, we could compare responses to a stimulus that contained only absolute disparity (the plane condition) to one that also contained relative disparity (the grating condition). Our spectral filtering approach separated time-courses that largely split the response into relative-disparity sensitive (odd harmonics) and relative disparity insensitive (even harmonics) response components. The even harmonic waveforms show an onset-time of ~ 75 ms for both plane and grating stimuli, while the robust odd-harmonic waveform derived from the grating response had an onset time of ~ 100 ms, *e.g*. ~ 25 ms later. Thus, the positive peaks at ~ 100 ms in the full waveforms in Fig. 3 come from the even-harmonic, transient response component. Previous VEP studies have uniformly reported the presence of a negative going component after disparity onset or change^37–39^. The positive peak that we see here at ~ 100 ms has been only infrequently reported (Skrandies, 2001). This peak is small and may thus have been difficult to record with conventional signal averaging and low-channel count recording approaches. Our filtering approach thus provides robust access to this earliest stage of disparity processing, situating the onset of disparity responses to be within ~ 25 ms of the onset of cortical response to image contrast (see^40^ for detailed discussion).

The initial stage of disparity processing appears to be largely unaffected by the presence of relative disparities the display. Responses to the disparity grating stimulus which contains both absolute and relative disparities diverge from the response to the plane stimulus (absolute disparity only) at 158 to 172 ms*, e.g.* well after the initial disparity-specific response occurs. This pattern of delayed relative disparity selectivity is consistent with what is known from the functional anatomy of single-unit recordings in macaque. Relative disparities are extracted in macaque V2, V3/V3A, V4 and IT, but not in V1^13,14,41–43^. Human fMRI results also suggest that relative disparity begins to be extracted in V2 and V3, but not in V1^32^. One thus expects EEG responses driven by relative disparity to be temporally delayed with respect to responses driven by absolute disparity. The previous single-unit studies have focused on tuning for relative disparity and have not presented time courses for the onset of relative *vs* absolute disparity selectivity as we do here.

By filtering the raw responses into even and odd harmonic components, we see that the odd-harmonic filtered component is strongly selective for relative disparity present in the grating condition. Moreover, the odd harmonic component is delayed relative to the even component, consistent with the former being driven by the presence of relative disparity in the grating stimulus. The 25 ms difference in odd- *v*s even-harmonic response onset (compare between Figs. 5c and 5d) is thus consistent with the even harmonic response reflecting the processing absolute disparity which is present in both plane and grating stimuli and the odd harmonics reflecting the extraction of relative disparity which is unique to the grating stimulus.

Prior work has also indicated that odd-harmonic responses are likely to arise from mechanisms that are sensitive to relative disparity. Odd-harmonic responses are strongly dependent on the availability of multiple disparities in the visual field^44–46^ consistent with the differences we see between plane and grating responses. Odd harmonic responses are tuned for cyclopean spatial frequency while even harmonics are not^35^, consistent with the even harmonics arising from mechanisms sensitive to absolute disparity. Here we add to these results by demonstrating that relative disparity is extracted after an initial transient response to absolute disparity that can be linked to even harmonic response components.

### Effects of task on disparity-specific responses

The response to the plane condition is dominated by even harmonic response components for which no modulation by task was seen. Because the stimulus isolates absolute disparity, the lack of a task effect is consistent with absolute disparity processing being task independent. What about the case of even harmonic/transient responses, more broadly? The grating stimulus has both absolute and relative disparity information and the response to the grating condition also contains even harmonics. Here the response is largely, but not entirely devoid of task effects. A small effect of task is measurable at around 230 ms after a disparity change. This modulation is small and occurs well after the larger, sustained task effect manifests in the odd-harmonic response.

Measurable odd-harmonic activity is present in the plane condition which is ~ 8 times smaller than what is recorded in the grating condition. Odd harmonic responses in the plane condition could arise from residual responses to incomplete isolation of relative disparity information from our display, from asymmetries in the response to direction of motion in depth or from asymmetries in the population response to zero and non-zero disparities. Some of this activity may be residual stimulus artefact that was not removed by the ICA filtering. The task effects that occur are at the times of disparity change and may therefore be due to wrap-around effects.

The largest and most striking task effects we measure arise for the grating stimulus and are brought to prominence through the ‘odd’ filter that emphasizes the VEP’s sustained response profile. We consistently found that the amplitude of the sustained negative-going potential was amplified when participants were attending to the stimulus directly. Thus, the observer’s task boosts neural responses to the relative disparity content of the grating stimulus, presumably via feedback from higher cortical areas. This modulation occurs at time points after the initial disparity encoding phases, from 167 ms onwards. The initial phase, from 75 to 158 ms is shared between grating and plane conditions, and is related to the initial extraction of absolute disparities. The period between 158–167 ms represents relative disparity coding that is pre-attentive. It is only after relative disparity has been extracted that the behavioral task is able to modify the neural response. Note that this time point may be too late to impact the transient responses seen either for the disparity plane, or for the even-filtered grating response. Alternatively, feedback connections may not target the anatomical substrate that generates the response to absolute disparity. These two explanations need not be mutually exclusive.

### Possible role of motor/pre-motor activity

A potential source of variation between conditions that could affect the VEP is motor or pre-motor activity arising from the button press task that was present during the “attend fixation” conditions but not the “attend stimulus” conditions. Any differences here could manifest during the sustained response where we attribute changes to the attentional state. However, button presses alone are not sufficient to induce a sustained response in the VEP, as can be seen in Fig. 4a where the button press task during the attend stimulus condition was not sufficient to induce a sustained response. Could motor/pre-motor activity instead modulate the response, contributing to the differences we see in Fig. 4b? We think this is unlikely for the following reasons. The task effect we observe is precisely time-locked to the onset of the disparity grating. Moreover, it occurs only at certain time-points, not being present at the earlier time-points of the disparity-evoked response. Motor or pre-motor activity is not time-locked to the disparity changes due to randomization of the presentation of the color change and random variations in reaction time. The expectation is therefore that motor or pre-motor activity will be uniform over the cycle average, and it will be small due to it occurring at random times with respect to the time-base of the stimulus.

Another source of variation could be changes in eye movements between experimental conditions, as suggested by previous studies showing that patterns of micro-saccades may differ under different task instructions^47^. Here, motor or pre-motor activity associated with micro-saccades could masquerade as a cognitive effect of attention. Pre-motor theory states that this motor preparation is both necessary and sufficient for attention to shift to the stimulus and thus spatial attention and motor preparation share the same underpinnings.

To evaluate the patterns of eye-movements during our different tasks, we measured the vertical EOG (VEOG) and horizontal (HEOG) signals derived from electrodes positioned above and below (VEOG) and to the sides of the eyes (HEOG).

At the group level, we observed no systematic, time-locked activity on the HEOG or the VEOG channels that could influence the time-locked attention effects (see Supplementary Figs. 1 and 2).

To quantify the variability of eye movements made during trials, we calculated the standard deviation of each participant’s mean HEOG and VEOG traces and compared variance within between attention conditions within a disparity type (see supplementary Figs. 3 and 4). There were no differences in HEOG or VEOG variability between tasks in the plane conditions. However, for the grating stimulus, we measured a 10% difference between the ‘attend fixation’ vs. ‘attend stimulus’ conditions for the HEOG signal (see Sect. 2 in Supplementary Materials for significance tests and Bayes Factors).

To determine if this difference was related to the VEP attention effect, we computed an eye movement modulation index and an VEP attention modulation index. Both indices were computed by taking the absolute difference of the variance between attention conditions and dividing by the sum (see Eq. 1 in the Supplementary Materials). These indices were uncorrelated and eye movements could not predict the magnitude of the attention effects (Supplementary Figs. 5–8).

Pre-motor theories of attention predict a strong relationship between micro-saccades and neural modulation in attention tasks, but recent work in macaque and human^48–51^ has shown that deployment of covert spatial attention (not unlike what we ask our participants to do—fixate centrally and spread attention globally) can produce modulations in the absence of micro-saccades.

Given the small size of the eye-movement differences, the lack of their correlation with neural modulation and indications from the prior literature of a non-causal relationship of microsaccades and neural modulation, we favor an explanation that the task effects we observe on the sustained response component are predominantly cognitive rather than oculomotor in origin.

### Cognitive penetrability of disparity processing

Our data speak to a conceptual framework that is prominent in the attention literature—the notion of cognitive penetrability. Cognitive penetrability and its converse, cognitive impenetrability, relate to the question of how early in the visual pathway the effects of the observer’s task manifest. A substantial body of evidence, largely derived from the ERP literature, has suggested that the earliest stage of cortical processing is not subject to task effects. This issue has been studied by an examination of the leading edge of the evoked response to the onset of an image which manifests as a component (C1) that occurs over ~ 50–80 ms. Image onset creates contrast, proto-object (see following section in Discussion), and object-level transients, but the leading edge reflects the initial extraction of contrast. While some studies have reported task effects on C1^52–54^ , other studies have indicated that C1 is not cognitively penetrable (see^55,56^ for reviews).

Here we also see an early response component—a positivity that arises at ~ 75 ms after disparity onset that too is cognitively impenetrable. This activity is seen in both plane and grating conditions and is associated with even response harmonics that show only small effects of task a much later time-points.

### The role of “proto-objects” in attention

The processing sequence we observe suggests the following sequence of coding stages: two task-independent coding stages, the first starting at ~ 75 ms involving the registration of absolute disparity which is followed by a brief period of task-independent coding of relative disparity. Task effects manifest after this point, but primarily in the odd-harmonic component of the disparity grating stimulus. This sequence of processing resembles the set of stages posited by psychophysicists from behavioral experiments^57–59^. Within this framework, the initial processing of a scene involves the rapid extraction of luminance, contrast, and edge information, as well as three dimensional surface orientation, and groupings of related edge fragments, resulting in the formation of a “proto-object” representation that does not depend on attention^57^, which is consistent with data from macaque V1 showing that enhancement of cell responses by image segmentation cues such as orientation contrast occurs in the absence of focused attention^11,24,60^. A similar pattern has also been observed in an Event-Related Potential (ERP) experiment on texture segmentation^61^.

A proto-object representation only loosely corresponds to everyday, recognizable objects and surfaces, but it goes considerably beyond coding of raw image statistics such as local orientation^58^ and here, absolute disparity. Attention, in this view, acts only after the formation of the proto-object representation. Relative disparity information fulfills the criteria required as the basis of a proto-object representation, as it can readily support the segmentation of figure and background and the computation of 3D surface orientation. Moreover, a relative disparity representation is largely independent of fixation distance, providing a degree of invariance in terms of form-from-disparity that would be useful for downstream invariant representations.

In our hands, the observer’s task modulates the response only once a relative disparity, *e.g.* a proto-object representation, has been established. In the case of texture-defined form processing in macaque V1, the representation of boundaries and surfaces defined by an orientation discontinuity were seen to be an automatic process, with attention acting only after these figure/group processes had been established^24^. Effects of task in an earlier study were also only seen at late time points, well after segmentation-dependent responses occurred^11^. Our results for absolute and relative disparity also suggest a hierarchy of proto-object representations that transitions from being task-independent to task-dependent.

Task and relative disparity extraction also appear to interact in the opposite direction—task can influence the speed of relative disparity processing (see Fig. 3). This two-way interaction between attention and proto-object formation has been previously observed in the case of the coding of border-ownership in macaque V2^26^. Differential cell responses to figure and background regions (*e.g.* the border ownership signal) manifested this distinction more rapidly when attention was directed to the cell’s preferred vs non-preferred stimulus, indicating an interaction between task and the extraction of a proto-object representation.

Other work on human disparity processing is consistent with the multi-stage model of proto-object formation and attention. By recording differences in ERPs between flat, zero disparity images and 3D images, the presence of disparity was found to modulate the response starting at 100 ms, but it was not until 150 ms that the magnitude of the difference potential predicted behavioral reaction time^40^. The corresponding value for the onset of disparity sensitivity in the current study is ~ 75 ms, and the onset of a task effect is 165–184 ms. Importantly, both studies have found a relatively long period when a disparity-specific response has begun but is not modulated by task or task performance.

### Conclusion

By using stimuli that have a hierarchy of disparity cues, from locally accessible absolute disparities to non-local relative disparity, we have demonstrated a hierarchy of processing leading to the formation of a proto-object representation. This representation is based on relative disparity and is cognitively penetrable.

## Methods

### Participants

24 participants (14 female, mean age 30 years) were recruited from the Stanford community. They all had normal or correct-to-normal visual acuity with no history of ocular diseases and neurological conditions. Visual acuity was assessed with a Bailey-Lovie LogMAR chart (Precision Vision, Woodstock, IL) with acuity of 0.1 LogMAR or better in each eye, and less than 0.3 LogMAR acuity difference between the eyes. The RANDOT stereoacuity test (Stereo Optical Company, Inc., Chicago, IL) was used to test stereo-acuity with a passing score of at least 50 arc seconds. Four participants were excluded from analysis, three due to low dot update responses (see below, Methods: Stimuli and Methods: VEP Signal Processing and Reliable Components Analysis) and one due to technical issues during recording. Data from 20 participants were retained for analysis. Participants’ informed written consent was obtained before experimentation under a protocol approved by the Institutional Review Board of Stanford University. All methods were performed in accordance with the relevant guidelines and regulations and in accordance with the Declaration of Helsinki.

### Visual display

Dynamic random-dot stereograms (DRDS) were displayed on a SeeFront 32″ autostereoscopic 3D monitor with a TFT LCD panel display in which a lenticular lens system presents separate image data to the left and right eyes. The SeeFront monitors participant position in front of the screen with an integrated pupil location tracker, and the eyes’ separate images are merged via line-interleaving in real time to form a single 3D image from the viewpoint of the participant. Viewing distance from the screen was 70 cm, within the optimal range as per the manufacturer for typical adult inter-pupillary distances. The screen has a native resolution of 3840 × 3160 pixels (1920 × 1080 effective binocular resolution) and was refreshed at 60 Hz. Mean luminance was 50 cd/m^2^.

### Stimuli

DRDS stimuli were like those described in detail elsewhere^35^. An example of side-by-side binocular half-images is shown in Fig. 1B. In brief, circular DRDS half-images (radius = 13.65°) were constructed of non-overlapping, 100% contrast black and white dots (15 dots/degree^2^, dot size 6 arcmin diameter) on a mean gray background. Dot positions were pseudorandom and regenerated in new locations at 20 Hz (every 3rd video frame). DRDS stimuli alternated at a rate of 1.4 Hz between ‘disparity on’ and ‘disparity off’ states (modulation depicted in Fig. 1c). During the ‘disparity on’ phase, stimuli generated a percept of a flat plane at 6 arcmin crossed disparity (‘plane stimulus’), or a percept of a horizontally-oriented, sinusoidal disparity grating (0.5 cpd) where binocular dot pairs were shifted systematically from being perfectly correlated (0 arcmin disparity) at the trough of the disparity grating, to being horizontally offset to generate 6 arcmin disparity at the peaks of the grating (see also Fig. 1a for a depiction of absolute disparities in the plane stimulus in both ‘disparity off’ and ‘disparity on’ states—points 1 and 2—and relative disparities in the grating stimulus—differences between points 1 and 2). The plane stimulus contained only absolute disparities, whilst the grating stimulus contained both local absolute and global relative disparities that defined the sinusoidal grating. During the ‘disparity off’ phase of the stimulus cycle, dot pairs were perfectly correlated between the eyes, generating a percept of a flat plane at zero disparity. Thus, our DRDS stimuli generated Visual Evoked Potentials at two fundamental frequencies, F1 (1.4 Hz) and F2 (20 Hz), as well as their harmonics, *n*F1 and *n*F2. *n*F1 were multiples of the 1.4 Hz disparity change, and thus were the frequencies of interest for judging the disparity effects. *n*F2 were multiples of the 20 Hz dot update response, providing an index for overall responsiveness to the dots in the DRDS. These stimulus-driven, frequency-tagged EEG responses were useful in later filtering stages (see Methods: VEP Signal Processing and Reliable Components Analysis).

**Figure 6:**
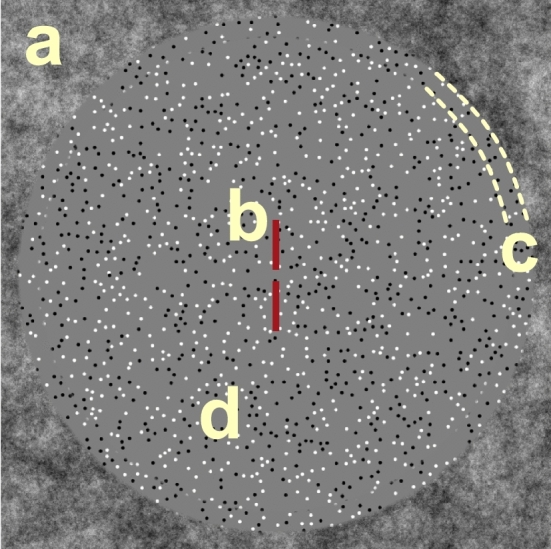
Schematic illustration of the DRDS display. A binocular, zero-disparity frame comprising 1/f noise (**a**) was used as a fusion lock. Dichoptic nonius lines (**b**) were used to engage fixation at the center of the screen and to monitor vergence. A detection task in which the color of the nonius lines changed from red to blue occurred at random intervals during both ‘attend fixation’ and ‘attend stimulus’ conditions. It was task relevant only in the ‘attend fixation’ condition. The duration of the color change was varied on a staircase which maintained an 82% detection rate. To minimize the availability of disparity references, a 1-degree gap containing binocularly uncorrelated dots (**c**) was interposed between the changing disparity region (**d**) and the fusion lock (**a**).

Figure 6 shows a schematic view of key elements in the stimuli. The DRDS was windowed using a 1/f fusion lock (39.5° square) presented in the periphery and at zero disparity, and which was static throughout each stimulus presentation and stabilized vergence eye position (Fig. 6, ‘a’). There was a band of uncorrelated dots between the fusion lock and the DRDS stimulus (1.2° wide; Fig. 6, ‘c’) which was sufficient to disrupt edge and binocular reference effects between the fusion lock and the disparity stimulus^62^. Centrally placed nonius lines (Fig. 6, ‘b’) were vertically separated and thus conveyed no horizontal disparity information, and together covered 1° of the stimulus vertically.

### Task and procedure

Two task conditions (Fig. 1c) were tested on each of the two stimulus types. In the first condition, participants were instructed to press a button when a color change of the nonius lines occurred and were instructed to solely attend to this central fixation task (“ignore the dots”). The duration of the color change was varied (between 0.5 and 0.01 s) using a staircase that maintained an 82% correct detection rate. Behavioral responses were tracked using the left arrow key on a keyboard. The DRDS stimuli were task-irrelevant in this condition. Color changes occurred at random times during the trial, with randomization occurring at several levels. The first color change occurred at random 2 to 3 s after the DRDS image appeared on the screen. The “clock” for the next color change was reset by the button press in response to the previous color change or 2 s, whichever was sooner. Thus, subsequent color changes also occurred at random times between approximately 2.5 to 5 s after the previous color change, depending on the reaction time. Finally, because the duration of the probe was on a staircase, an additional degree of randomization of the inter-probe interval was present. In practice there were two to three color changes per 10-s trial.

In the second condition, participants were instructed to attend to the DRDS stimulus while maintaining central fixation at the nonius lines (“fixate centrally but spread your attention to the dots”) and were asked to ignore the ongoing color change of the nonius lines. The DRDS stimuli were thus task-relevant in this condition. Each task condition was tested with both disparity plane and grating displays for a total of four experimental conditions (plane stimulus, attend fixation; plane stimulus, attend stimulus; grating stimulus, attend fixation; grating stimulus, attend stimulus). Condition-specific instructions were verbally given at the start of each set of 10 trials, and sets were always of the same condition type.

Each stimulus trial lasted 12 s and was composed of a 1 s dynamic prelude to allow the EEG to achieve steady-state, followed seamlessly by a 10 s active task block, and ending with a 1 s dynamic postlude. The prelude and postlude recycled the first and last 60 frames of the stimulus presented during the active task block, respectively. Participants were instructed to blink as needed during jittered inter-trial intervals (1500 ± 500 ms). Each of the four conditions were presented in sets of 10 trials. Each condition set was repeated 3 times for a total of 30 trials per condition. The order of the sets was randomized and participants were permitted to rest between sets.

### EEG acquisition and pre-processing

The EEG was recorded with 128-channel HydroCell sensor nets and an Electrical Geodesics Net Amps 400 amplifier (Electrical Geodesics, Inc., Eugene, OR, USA). The EEG was sampled natively at 500 Hz and then resampled at 420 Hz, giving 7 data samples per video frame. EEG data were first bandpass filtered from 0.3 to 50 Hz, then evaluated according to a sample-by-sample thresholding procedure to identify consistently noisy individual sensors. These channels were interpolated by six neighboring channels. Once noisy sensors were substituted, the EEG was re-referenced from the Cz reference to the average of all 128 electrodes. Finally, 1 s EEG epochs containing a large percentage of data samples (> 10%) exceeding threshold (30 to 80 mV) were excluded on a sensor-by-sensor basis. These epochs were typically associated with eye movements or blinks. Some of the individual participants (n = 8) had a systematic artifact arising on electrodes that had shown high 60 Hz power line noise during the recording. This artifact was characterized by sharp, clearly non-biological deflections of the EEG signal at regular intervals associated with the digital input trigger and were identifiable based on a characteristic topography, waveform, and Fourier spectrum. ICA was used to remove these artifacts, when present After running ICA on a given participant, we visually inspected the results (topographies, waveforms and, Fourier spectra of the ICA components) and identified 8 datasets which contained components with all the above-described signatures. In these 8 participants we removed the components (usually one to two) that were related to the artifact.

### VEP signal processing and reliable components analysis

**Figure 7:**
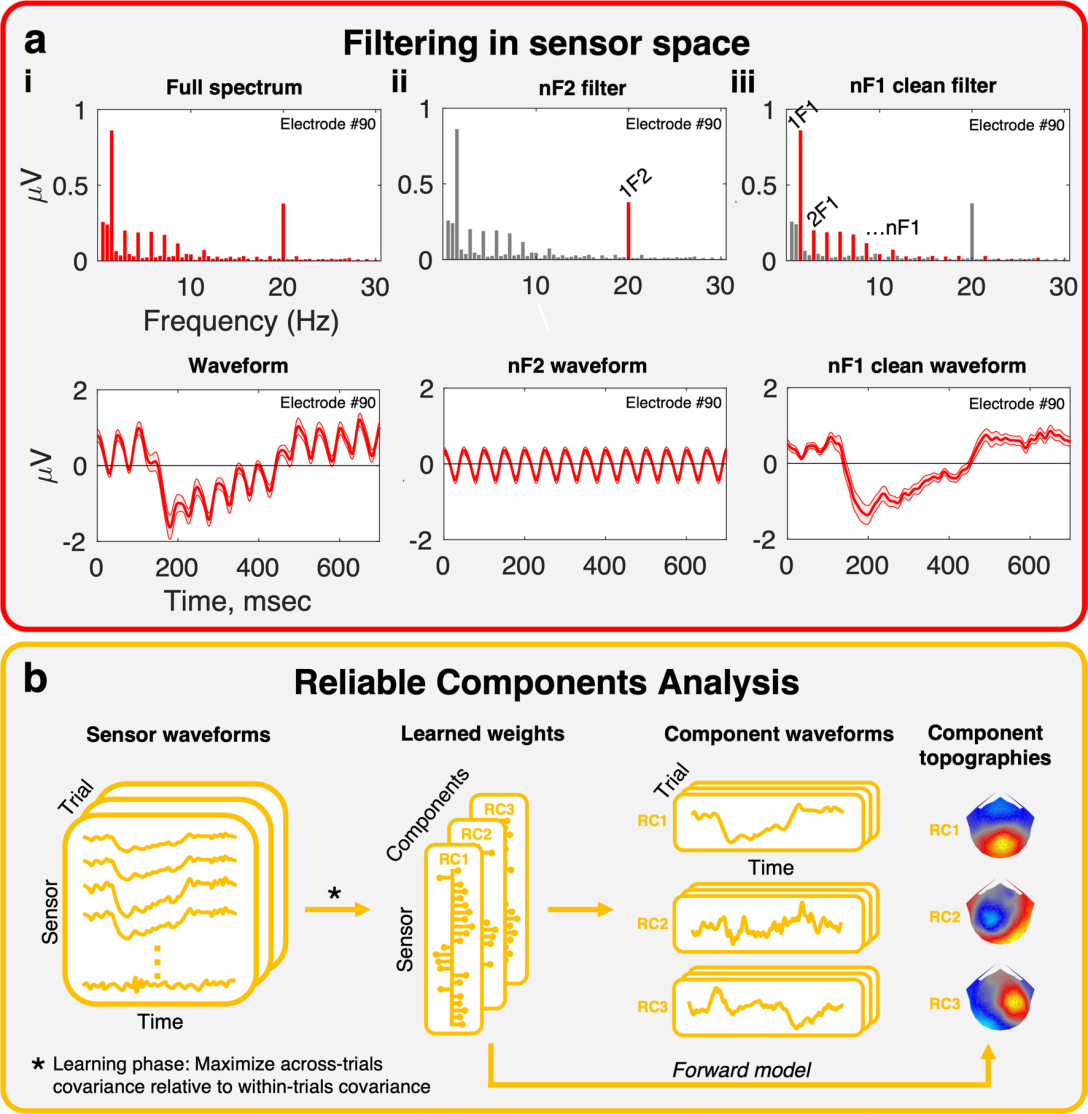
Two key stages in the data processing pipeline. (**a**) Filtering in sensor-space. Fourier pairs (spectra on the top row, and corresponding waveforms on the bottom row) illustrate different filter types. The full spectrum, **a**(**i**), contains both signal and noise harmonics, dominated by signals at multiples of the fundamental frequencies in the stimulus. This signal can be filtered to highlight different response components. The nF2 filter, **a**(**ii**), removes all frequencies in the response except those at multiples of 1F2 (in our stimuli, the dot update rate at 20 Hz). The dot update can also be removed, and the disparity response highlighted, using the nF1 clean filter, **a**(**iii**). Here, multiples of the 1F1 fundamental (in our stimuli, disparity update at 2 Hz) are preserved and all other frequencies are removed. The reconstructed waveform is a “clean” version of the full waveform, with non-disparity and dot update signals removed. (**b**) Dimensionality reduction via Reliable Components Analysis. The nF1 clean data are provided as input to the RCA pipeline, where components are learned that maximise the across-trials covariance relative to the within-trials covariance. Components are vectors of electrode weights where electrodes that respond in a consistent manner across trials are emphasized. These learned weights can be projected through a forward model, revealing underlying neural sources in the form of component topographies. Trial data are projected through the weight vectors to generate mean responses for each component, reducing the dimensionality of the data from 128 sensors to a small number of reliable components. Typically RCA is run on the group level, where input data are individual participant trials but weights are learned across all participants, resulting in group-level topographies and group-level waveforms.

The first step in our signal processing pipeline (Fig. 7a) was to separate responses to the 20 Hz dot update rate from responses to the 1.4 Hz disparity change. This was done using the Fourier transform as a filter. Panel a(i) shows the spectrum of the response to a disparity grating in the top row and the corresponding time average response in the bottom row, reconstructed via an inverse Fourier transform. Panel a(ii) isolates the dot update response at 20 Hz (1F2) and 40 Hz (2F2, not shown). Panel a(iii) removes the dot update response, leaving only the harmonics of the disparity response (nF1) that do not overlap with the dot update rate in the reconstruction on the right. We also used the Fourier transform to filter the data into odd (1F1, 3F1, 5F1, etc.) and even (2F1, 4F1, 6F1, etc.) harmonic components as these have been found to have different functional properties^35^.

The second step in the processing (Fig. 7b) was to reduce the dimensionality of the data using Reliable Components Analysis (RCA) (Dmochowski et al., 2015). RCA is a spatial filtering approach akin to PCA, with the distinction that components are identified based on maximizing trial-by-trial covariance rather than variance explained^63^. RCA computes linear combinations of electrode weights to maximize correlation, in time, of data across all trials of the same experimental condition. The optimization procedure, described in^27,64^, reduces to an eigenvalue solution where the resulting weight vectors and their coefficients are the ordered eigenvectors and eigenvalues, respectively, of across-trial covariance relative to within-trial covariance^65^. Thus, EEG data are decomposed from electrode-by-time matrices to component-by-time matrices, and components are sorted in descending order to maximize correlation in the first component. Finally, weight vectors are passed through a forward model^27,28^ to reveal physiologically plausible topographies that indicate the neural sources likely to underlie the signal. For our purposes, we trained two sets of RCA filters on filtered data with the dot update response removed (‘nF1 clean’ filter), and components were learned across task conditions but within each disparity stimulus type. Our approach yielded two sets of weights representing the response to plane or grating type stimuli (forward models shown in Fig. 2). In both cases the first reliable component, RC1, was dominant and subsequent components did not contain significant signals, thus we only present data from RC1.

### Permutation-based statistical analysis

Timepoints where EEG responses from two different conditions differed from one another were localized using a permutation-based, paired-samples *t-*test which intrinsically corrects for multiple comparisons^66–68^. Synthetic datasets were generated from the EEG data by randomly permuting condition labels across participants and calculating pairwise *t*-scores of the resulting waveform differences. For each permutation, we identified the longest consecutive run of time points where *p*-values were less than 0.050, generating a non-parametric reference distribution of significant consecutive timepoints across permutations. Consecutive trains of significant *t*-scores in the original, non-permuted data that exceeded 95% of the values in the reference distribution were considered statistically significant, and such epochs are marked with asterisks (Figs. 3, 4 and 5). Since this procedure is dependent on both the length of the data and the arbitrary 95% threshold, we also present the uncorrected significance values (heat bars in Figs. 3, 4 and 5).

To aid in the interpretation of resultant *p* values we computed the mean Bayes factor between timepoints of interest that were statistically significant or non-significant, providing a ratio of the likelihood of ${H}_{1}$ to the likelihood of ${H}_{0}$. This analysis was carried out using a MATLAB toolbox^69^ that is available at https://github.com/klabhub/bayesFactor. We interpret the Bayes factor with reference to evidence categories suggested in^33,34^ though these should be treated as approximate descriptors only^33^ and are more meaningful in the context of results from the run-length corrected permutation *t*-test, as well as in relation to one another. In some apparently conflicting instances, we measure Bayes factors that would –by themselves—be considered moderate evidence for rejecting the null hypothesis but where results from the permuted *t*-test do not support this. In such cases the measured Bayes factors are also many orders of magnitude smaller than those measured at different timepoints. Here, we preference the results from the permutation *t*-test since this intrinsically corrects for multiple comparisons, whilst our calculation of the Bayes factor includes those time periods where the alpha threshold of *p* ≤ 0.050 is surpassed but the run-length criterion is not. The mean Bayes factor we report is influenced by these outlier timepoints.

The results of the permutation t-tests are useful in identifying a timeline of events—for example, comparing the time at which responses to different stimuli differentiate under different attentional conditions (see Fig. 3 for an example). To quantify such comparisons, we used a Jackknifing approach^70–72^ where we iteratively re-calculated the permutation *t*-tests in a leave-one-participant-out analysis. With each iteration, this analysis returned the timepoint at which two conditions significantly diverged from one another—for this, we took the first timepoint in the first segment that survived the run-length correction. Thus, the Jackknife procedure returned a distribution of “onset-of-the-effect” timepoints from which we calculated 95% confidence intervals, and these distributions were finally compared with a *t-*test.

## Supplementary Information

Supplementary Information.

## Data Availability

The datasets used and/or analysed during the current study available from the corresponding author on reasonable request.
