# Supplementary material for: Cognitive penetrability of scene representations based on horizontal image disparities
Source: Sci Rep. 2022 Oct 25;12:17902. doi: 10.1038/s41598-022-22670-7 (PMC9596438; doi:10.1038/s41598-022-22670-7)
Supplement: Supplementary file 1 — Supplementary Information. [file 41598_2022_22670_MOESM1_ESM.docx]

**Supplementary Materials**

**Measuring task- and stimulus-related eye movements via HEOG and VEOG**

We measured the vertical electro-oculogram (VEOG) and horizontal electro-oculogram (HEOG) signals that can be derived from electrodes positioned above and below (VEOG) and to the sides of the eyes (HEOG). Because our high-density EEG net includes these electrode placements, we were able to record and analyse horizontal and vertical eye movements synchronously with the visual responses we reported from posterior visual areas. The estimated precision of EOG for fixation monitoring is ~ 0.5 degrees ^1^.

**1: Are there time-locked eye movements?**

We calculated group-level HEOG and VEOG cycle averages to visually check for time-locked activity that could influence the attention effects we describe in the main manuscript. These averages are synchronized to the disparity change stimulus in the same way that our VEP analyses are and have the same time-base and sampling rate. Figures are shown below. Because these traces are flat to within the variance of the measurement, our conclusion from the plots below is that there is no time-locked activity on the HEOG. There is however a time-locked signal on the VEOG. This activity is contamination from volume-conducted dot update and disparity VEPs from posterior visual areas and is not the vertical eye movement signal per se.

To remove VEP contamination, we detrended subject-level data in subsequent analyses by subtracting the group level mean from each trial on a condition-by-condition basis. We did this for both the HEOG and the VEOG.

**Supplementary Figure 1:** HEOG cycle averages for all stimulus types and all stimulus conditions. Cycle averages are group-level means and the shading indicates the SEM across subjects.

**Supplementary Figure 2:** VEOG cycle averages for all stimulus types and all stimulus conditions. Cycle averages are group-level means and the shading indicates the SEM across subjects.

Note that the error on the traces indicates the cross-subject variance (SEM) – i.e. differences across subjects in the HEOG / VEOG. A better measure of the relevance of eye movements to the VEP responses is the within-subject variance on detrended HEOG and VEOG traces, described next.

**2: Does eye movement variability differ between attention conditions?**

We calculated individual participant cycle averages after removing the group mean from individual trial data (detrending), for each condition separately. We next calculated the standard deviation of each participant’s HEOG and VEOG trace, giving an indication of the magnitude of HEOG and VEOG for each participant, in each condition. Plots below show the detrended cycle averages for each participant, and the values in the key are the standard deviation for each trace.

**Supplementary Figure 3:** Detrended HEOG traces for each individual subject. Traces are cycle averages across all trials, and the key denotes the variance (standard deviation) of each trace, i.e. the magnitude of the variability in the HEOG for each subject. These measures were entered into a paired samples t-test to test for differences in the HEOG between attention conditions, within a stimulus type.

Standard deviation values were entered into a within-subjects t-test comparing across attention conditions, within each stimulus type. For plane stimuli HEOG, there were no differences in variability between attend fixation and attend stimulus conditions (mean attend fixation = 0.41, sem = 0.03; mean attend stimulus = 0.47, sem = 0.03; t(19) = -1.79, *p* = .090, bf_10_ = 0.89 – anecdotal evidence for H_0_). For grating stimuli HEOG, there was a difference in variability where the variance in HEOG for the attend stimulus condition was, on average, ~10% larger than in the attend fixation condition (mean attend fixation = 0.40, sem = 0.02; mean attend stimulus = 0.45, sem = 0.03; t(19) = -2.82, *p* = .011, bf_10_ = 4.76 – moderate evidence for H_1_). This implies that participants made larger horizontal eye movements in the attend stimulus condition, when viewing grating stimuli.

**Supplementary Figure 4:** Detrended VEOG traces for each individual subject. Traces are cycle averages across all trials, and the key denotes the variance (standard deviation) of each trace, i.e. the magnitude of the variability in the VEOG for each subject. These measures were entered into a paired samples t-test to test for differences in the VEOG between attention conditions, within a stimulus type.

For VEOG, we found no significant differences for either plane (mean attend fixation = 0.32, sem = 0.03; mean attend stimulus = 0.31, sem = 0.02; t(19) = 0.89, *p* = .383, bf_10_ = 0.33 – moderate evidence for H_0_) or grating (mean attend fixation = 0.36, sem = 0.04; mean attend stimulus = 0.36, sem = 0.04; t(19) = 0.11, *p* = .913, bf_10_ = 0.23 – moderate evidence for H_0_) stimulus conditions, indicating that the vertical EOG was similar between attention conditions within each stimulus type.

We found that, whilst VEOG and HEOG were similar between attention types for most conditions, there was a difference in the variability of HEOG when participants were viewing grating stimuli. Although this difference was small, it was statistically reliable and implies that larger horizontal eye movements are made when attending the modulation of a relative disparity grating. Since this is the condition where we measure our attention effects, specifically on the sustained response component, we probed whether these eye movements are related to the magnitude of the attention effect.

**3: Does eye movement variability correlate with the attention effect?**

We calculated an ‘eye movement index’ (Equation 1) and an ‘attention modulation index’ for each participant on the basis of HEOG and VEOG variance in the former case, and variance in the cycle averages for different attention conditions in RC1 in the latter case. The ‘eye movement index’ I_EM_ was computed by

$$I_{EM}= \frac{2(|\sigma_{t}^{1}-\sigma_{t}^{2}|)}{\sigma_{t}^{1}+\sigma_{t}^{2}}$$

Equation 1

where $\sigma_{t}^{1}$ was the standard deviation of the HEOG (or VEOG) in the attend fixation condition, and $\sigma_{t}^{2}$ was the standard deviation of the HEOG (or VEOG) in the attend stimulus condition. The ‘attention index’ was calculated in the same manner, expect now $\sigma_{t}^{1}$ and $\sigma_{t}^{2}$were the standard deviation of the mean EEG cycle average in RC1 during the attend fixation and the attend stimulus conditions. Indices were then plotted on a scatter plot (shown below) and entered into a linear model to test for a relationship between the extent of HEOG and VEOG variability, and the nominal attention effect. Plots are results from the linear model fits are shown below. To summarize, We did not find any evidence that the attention modulation index could be predicted from the eye movement index, even in the HEOG grating conditions, implying that these phenomena are separate.

**HEOG, plane conditions:**

****The linear regression model found no relationship between the eye movement index and the attention index, R^2^ = 0.01, F(1,18) = 0.12, *p* = .731

**Supplementary Figure 5:** Scatter plot showing the relationship between the magnitude of the variability in the HEOG versus the magnitude of the attention effect for the plane stimulus. Each dot represents data from 1 subject, and the red line is the result of the linear model fit.

**HEOG, grating conditions**

The linear regression model found no relationship between the eye movement index and the attention index, R^2^ = 0.004, F(1,18) = 0.08, *p* = .779****

**Supplementary Figure 6:** Scatter plot showing the relationship between the magnitude of the variability in the HEOG versus the magnitude of the attention effect for the grating stimulus. Each dot represents data from 1 subject, and the red line is the result of the linear model fit.

**VEOG, plane conditions:**

The linear regression model found no relationship between the eye movement index and the attention index, R^2^ = 0.01, F(1,18) = 0.21, *p* = .650

**Supplementary Figure 7:** Scatter plot showing the relationship between the magnitude of the variability in the VEOG versus the magnitude of the attention effect for the plane stimulus. Each dot represents data from 1 subject, and the red line is the result of the linear model fit.

**VEOG, grating conditions:**

The linear regression model found no relationship between the eye movement index and the attention index, R^2^ = 0.03, F(1,18) = 0.03, *p* = .875

**Supplementary Figure 8** Scatter plot showing the relationship between the magnitude of the variability in the VEOG versus the magnitude of the attention effect for the grating stimulus. Each dot represents data from 1 subject, and the red line is the result of the linear model fit.

Overall, although we do measure a significant difference in variability of the HEOG between attend stimulus and attend fixation for the grating stimulus conditions, we cannot detect that these effects are related to the modulation in the EEG as participants switch between these two tasks. Therefore, we favour our interpretation that this modulation in the EEG is indeed caused by cognitive factors (attention) and is not an artifact of eye movement differences.

**References:**

1 Verghese, P., Kim, Y. J. & Wade, A. R. Attention selects informative neural populations in human V1. *Journal of Neuroscience* **32** (2012).
